# Differential hRad17 expression by histologic subtype of ovarian cancer

**DOI:** 10.1186/1757-2215-4-6

**Published:** 2011-03-30

**Authors:** Jennifer L Young, E Colin Koon, Joseph Kwong, William R Welch, Michael G Muto, Ross S Berkowitz, Samuel C Mok

**Affiliations:** 1 Division of Gynecologic Oncology, Department of Obstetrics, Gynecology and Reproductive Biology, Brigham and Women's Hospital, Harvard Medical School, Boston, MA, USA; 2 Division of Gynecologic Oncology, Department of Obstetrics and Gynecology, Medical University of South Carolina, Charleston, SC, USA; 3 Division of Gynecologic Oncology, Department of Obstetrics and Gynecology, Baylor University Medical Center, Dallas, TX, USA; 4 Centre for Translational Oncology, Institute of Cancer and the CR-UK Clinical Centre, Barts and the London Queen Mary's School of Medicine and Dentistry, London, UK; 5 Department of Pathology, Brigham and Women's Hospital, Harvard Medical School, Boston, MA, USA; 6 Department of Gynecologic Oncology, University of Texas MD Anderson Cancer Center, Houston, TX, USA

## Abstract

**Background:**

In the search for unique ovarian cancer biomarkers, ovarian specific cDNA microarray analysis identified hRad17, a cell cycle checkpoint protein, as over-expressed in ovarian cancer. The aim of this study was to validate this expression.

**Methods:**

Immunohistochemistry was performed on 72 serous, 19 endometrioid, 10 clear cell, and 6 mucinous ovarian cancers, 9 benign ovarian tumors, and 6 normal ovarian tissue sections using an anti-hRad17 antibody. Western blot analysis and quantitative PCR were performed using cell lysates and total RNA prepared from 17 ovarian cancer cell lines and 6 normal ovarian epithelial cell cultures (HOSE).

**Results:**

Antibody staining confirmed upregulation of hRad17 in 49.5% of ovarian cancer cases. Immunohistochemistry demonstrated that only 42% of serous and 47% of endometrioid subtypes showed overexpression compared to 80% of clear cell and 100% of mucinous cancers. Western blot confirmed overexpression of hRad17 in cancer cell lines compared to HOSE. Quantitative PCR demonstrated an upregulation of hRad17 RNA by 1.5-7 fold. hRad17 RNA expression differed by subtype.

**Conclusions:**

hRad17 is over-expressed in ovarian cancer. This over-expression varies by subtype suggesting a role in the pathogenesis of these types. Functional studies are needed to determine the potential role of this protein in ovarian cancer.

## Background

Ovarian cancer is the most deadly of all gynecologic malignancies[1]. Because ovarian cancer is diagnosed in Stage III or IV in 80% of cases, the prognosis is poor with only a 44% overall survival rate[1]. However, when ovarian cancer is diagnosed in the earliest stage, survival approaches 90%. Therefore we continue to search for better methods of early detection and for new therapeutic targets. Microarray technology allows the simultaneous comparison of two different populations to identify unique gene expression profiles. Use of microarray technology has been instrumental in identifying new genes possibly involved in the pathogenesis of ovarian cancer as well as secretory proteins that may have clinical utility as serum markers[2-5]. An ovarian-specific complementary DNA (cDNA) chip showed differential expression of hRad17 in cancer cells of long term survivors compared to short term survivors with Stage IIIC ovarian cancer[6].

hRad17 is the human homologue of a cell cycle checkpoint protein that was originally identified in yeast and is normally expressed in the human testis[7,8]. This protein is involved in DNA damage recognition and repair and is associated with accumulation of p53[9]. hRad17 is a nucleolar protein that disperses after DNA damage[10,11] and is activated by ATR-mediated phosphorylation[12-15]. This protein interacts with DNA polymerase ε [10] and serves as the clamp loader for the hRad 9-1-1 sliding clamp polymerase[16-19]. Both mechanisms are important for G2 checkpoint during cell replication. Recent data also demonstrates an interaction with DNA ligase I[20]. Loss of the function of hRad17 or aberrant expression may lead to malfunction of DNA repair and ultimately the development of cancer[21,22]. Alternatively, elevated expression in the setting of cancer may lead to increased resistance to DNA-damaging agents. These checkpoint proteins may serve as important therapeutic targets[23].

Prior studies have shown that hRad17 is upregulated in other cancers including colon, breast, and lung cancer[7,14,24] but no studies have thus far been conducted in ovarian cancer. The aim this study is to validate the over-expression of hRad17 in ovarian cancer and correlate this data with clinical outcomes.

## Methods

### Cell lines and tissue samples

All patient-derived specimens were collected and archived under protocols approved by the Brigham and Women's Hospital Human Subjects Committee, Brigham and Women's Hospital, Boston, MA, or as an approved use of discarded human materials as previously described[4]. All 17 ovarian cancer cell lines (CaOV3, DOV13, MCAS, OVCA3, OVCA420, OVCA429, OVCA432, OVA433, OVCA633, PEO4, SKOV3, TOV21G, RMG-1, ES2, TOV112 D, RMUG-L, RMUG-S) and 6 human ovarian surface epithelium (HOSE) cultures (HOSE2105, HOSE2107, HOSE 2139, HOSE 2166, HOSE 2170, HOSE 2177) were obtained and grown in conditions as previously described[25]. RNA was extracted from individual or pooled cell lines by using micro RNA extraction kit as described by the manufacturer (Qiagen, Valencia, CA) and quantified by fluorometry (Gemini Bio-Products, Inc, Calabasas, VA.). Clinicopathologic information, including diagnosis, disease stage and grade, and months survival, was collected from the patients' charts. All pathologic samples were re-reviewed for confirmation of histologic type and diagnosis.

### RNA extraction and real-time quantitative polymerase chain reaction

Quantitative RT-PCR was performed on total RNA prepared from 11 serous, 3 clear cell, 1 endometrioid, and 2 mucinous ovarian cancer cell lines as well as 6 normal ovarian epithelial cell cultures. For the quantitative RT-PCR studies a total of 1 μL (0.1 μg) cDNA was used in a 25 μL PCR mix containing 1X SYBR PCR buffer, 3 mM MgCl_2_, 0.8 mM dNTP, and 0.025 U/μL AmpliTaq Gold (PE Applied Biosystems, Foster City, CA). Amplification was then performed in duplicate using primer sets purchased from Sigma GenoSys (The Woodlands, TX) (forward primer: 5'-TCCCTCTGAAGCGACACTTT-3', reverse primer: 5'-AGTGGCTTGAGTGGGTTCAC-3') and glyceraldehyde-3-phosphate dehydrogenase (GAPDH) for normalization of input RNA in an ABI PRISM 5700 Sequence Detector (PE Applied Biosystems). RT-PCR was run with denaturation for 10 minutes at 95°C then 40 PCR cycles of denaturation at 95°C for 15 seconds and finally annealing or extension at 60°C for 1 minute. The relative level of hRad17 for each sample was calculated as described[4]. In brief, the relative amount of PCR products generated from each primer set was determined on the basis of the *C_t _*value. GAPDH was used to normalize the quantity of RNA used. Its *C_t _*value was then subtracted from that of each target gene to obtain the Δ*C_t _*value. The difference between the Δ*C_t _*value and the calibrator (Δ*C_t _*of sample HOSE 21) was determined as the ΔΔ*C_t_*. The representative quantitative value was expressed as 2^-ΔΔ*Ct*^. The Mann Whitney U test for nonparametric data was used to compare the distributions of all cancer cell lines to normal HOSE as well as the serous cancer cell lines to normal HOSE using SAS software version 9.1.3 (SAS Institute Inc, Cary, NC)

### Western blot analysis

Western blot analyses were performed using ovarian tumor cell lysates from 4 ovarian cancer cell lines (ES2, PEO4, DOV13, and OVCA 429) and 2 normal ovarian epithelial cell cultures (HOSE 667 and HOSE 21) using the mouse monoclonal anti-hRad17 antibody (provided by Dr. Lan Bo Chen's laboratory at Dana Farber Cancer Institute, Boston, MA) previously described in immunohistochemistry on breast and colon tissues[7,24], and a rabbit monoclonal antibody against phosphorylated or activated hRad17 (Santa Cruz Biotechnology, Inc, Santa Cruz, CA). In brief, a total of 25 μg protein for each sample were electrophoresed on a 10% SDS-PAGE gel. They were then transferred to a PVDF membrane for 1 hour. Membrane was blocked overnight in 5% milk in washing buffer (TBST, created from 10 mL 1 M Tris, 20 mL of 5 M NaCl, and 1 mL Tween 20 to volume of 1 L) at 4°C and incubated manually with an anti-hRad17 or an anti-phosphorylated hRad17 using 1:1000 dilution or 5 μL in 5 mL 5% milk in washing buffer for 1 hour at room temperature followed by a wash in TBST for 45 min. The membrane was then incubated with secondary antibody (either goat-anti-mouse for anti-hRad17 or goat anti-rabbit for anti-hRad17-phos) 0.5 μL/mL in washing buffer. The membrane was then again washed for 45 min. Immunoreactivity was detected using the ECL Chemiluminescence System (Amersham, Piscataway, NJ). For normalization of protein loading, the same membrane was incubated with an anti-β-actin monoclonal antibody (Sigma, St. Louis, MO).

### Immunohistochemistry

Immunostaining was performed using a mouse monoclonal anti-hRad17 antibody as described above. Tissue sections were prepared from 72 serous, 19 endometrioid, 10 clear cell, and 6 mucinous ovarian cancer cases in addition to 9 benign ovarian epithelial tumors and 6 normal ovaries. The slides were first incubated at 60°C overnight. They were then deparaffinnized in xylene and rehydrated in graded ethanol. The slides were then washed in Tris-buffered saline (TBS) for 5 minutes. Blocking serum was made using 10 mL TBS with 1% bovine serum albumin (BSA). Slides were incubated with the blocking serum for 30 minutes and washed prior to incubation with anti-HRad17 antibody (2 μg/mL) for 1.5 hours. Slides were then washed in TBS for 20 min. The slides were then stained using the Universal DakoCytomation EnVision System-AP with Fast Red Substrate-Chromogen, and EnVision Labeled Polymer, Alkaline Phosphatase (DakoCytomation, Dako, Denmark) as described in the product's directions. First the slides were incubated with alkaline phosphatase labeled polymer for 30 minutes, washed in TBS for 20 min, and incubated with the Fast Red chromogen solution for 15 minutes. After washing in water, each slide was then mounted and the immunoreactivity was quantified using a semi-quantitative scoring system described previous[26]. A weighted score was obtained by multiplying the score (0-3) for intensity on a scale of 0 for no staining and 3 for maximum intensity of red staining and the score for percentage stained (0-4). For percentage stained 0 represented no staining of the specimen, 1 equal to less than 25% stained, 2 equal to 25-50% stained, 3 equal to 50-75% of specimen stained, and 4 equal to > 75% of specimen staining positive. A mean score was then established at 3. Scores ≥3 were considered positive for hRad17 expression and scores <3 were considered negative for expression. Scores were compared using Kruskal-Wallis analysis for nonparametric data. Representative photomicrographs were recorded by digital camera (Optronic, Inc., Muskogee, OK).

### Correlation with Patient Survival

Clinical survival data was obtained from 72 cases of Stage 3, Grade 3 serous ovarian cancer from 1990-2003 starting with the date of the initial operation to the most recent visit. 66 of 72 patients were deceased at the time that the charts were reviewed. Patients were further divided into optimally and suboptimally debulked patients as defined by <2 cm nodules of residual disease at the end of surgery. Survival data was correlated with hRad17 over-expression and results obtained were examined by Kaplan Meier survival analysis. Statistical significance was determined by the log rank test.

## Results

### Quantitative RT-PCR

**Figure 1:**
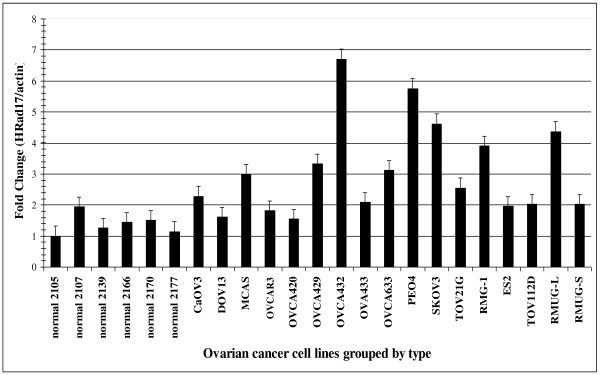
**RT-PCR showing hRad17 over-expression by ovarian cancer cell line compared to HOSE**. (TOV112 D is an endometrioid type ovarian cancer cell line.).

Quantitative real-time PCR was performed on total RNA prepared from 11 serous, 3 clear cell, 1 endometrioid, and 2 mucinous ovarian cancer cell lines as well as 6 normal ovarian epithelial cell cultures. RT-PCR demonstrated an upregulation of hRad17 RNA by 1.5-7 fold relative to HOSE. Fourteen of seventeen ovarian cancer cell lines showed up-regulation of hRad17 RNA compared to one of six normal ovarian epithelial cell cultures, p = 0.0013. In addition, PCR confirmed differential expression of hRad17 RNA by subtype with 2/2 mucinous and 3/3 clear cell cancer lines compared to 8/11 serous tumor cell lines. Mucinous and clear cell types all have 2-4 fold over-expression (Figure 1). While the mucinous and clear cell groups were too small for comparison, the serous cancer cell lines were also found to have a significantly different distribution of hRad17 compared to normal with p = 0.0091.

### Western blot Analysis

**Figure 2:**
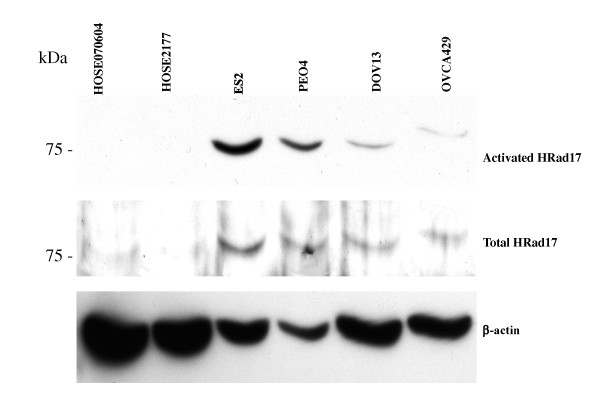
**Western blot of ovarian carcinoma cell lysates showing elevated expression of both total and activated hRad17**.

Next, western blot was used to confirm over-expression of total and phosphorylated hRad17 protein in 4 ovarian cancer cell lines compared to 2 normal HOSE cell lines. Three of four ovarian cancer cell lines were positive for over-expression of hRad17 compared to 0/2 normal HOSE. Further, ES2 and PEO4 were strongly positive for activated hRad17 and DOV13 and OVCA 429 were weakly positive for hRad17 expression compared to no expression in 2 normal HOSE cell lines (Figure 2).

### Immunohistochemistry

Immunostaining of hRad17 protein was performed using a mouse monoclonal anti-hRad17 antibody previously described in immunohistochemistry on breast and colon tissues[7,24]. Tissue sections were stained from 72 serous, 19 endometrioid, 10 clear cell, and 6 mucinous ovarian cancers in addition to 9 benign ovarian epithelial tumors and 6 normal ovaries. Over-expression was defined by a standard scoring system to judge the intensity and percentage stained and each slide was given a score of hRad17 expression by two independent reviewers who were blinded to the histologic type prior to review. Antibody staining confirmed upregulation of hRad17 in 53 of 107 (49.5%) ovarian carcinomas.

**Figure 3:**
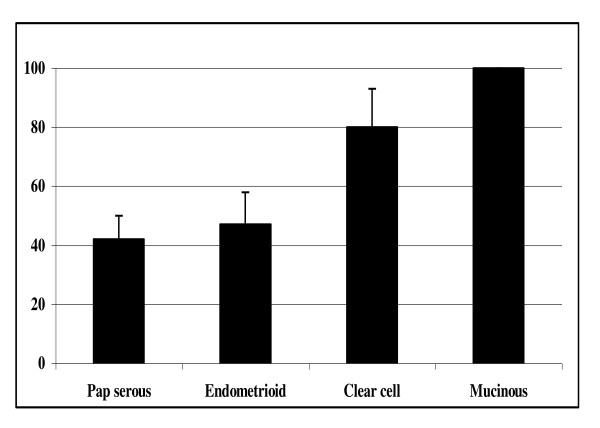
**Percentage overexpression hRad 17 determined by immunohistochemistry quantitative analysis comparing different subtypes of ovarian cancer**.

Immunohistochemistry further demonstrated differential expression among different subtypes of ovarian cancer. While only 42% (30/72) of papillary serous and 47% (9/19) of endometrioid subtypes showed over-expression, 80% (8/10) of clear cell and 100% (6/6) of mucinous tumors over-expressed hRad17 (Figure 3). hRad17 over-expression was significantly higher in mucinous and clear cell subtypes compared to serous cancer (p = 0.002, p = 0.005 respectively).

**Figure 4:**
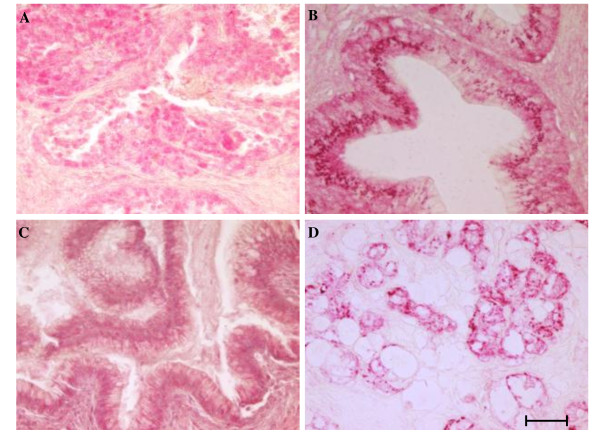
**Representative photomicrographs of hRad17 staining in different histologic types**. A papillary serous, B endometrioid, C mucinous, and D clear cell. Bar = 50 μm.

In addition, there were noted to be clear differences in the pattern of hRad17 expression between ovarian cancer and benign tissue as well as among the different subtypes of ovarian cancer (Table 1). While in benign ovarian epithelium hRad17 staining demonstrated a nuclear location only consistent with the positive controls, the papillary serous tumors exhibited hRad17 staining in the cytoplasm as well as in the nucleus. Mucinous cancers were also strongly positive in the nucleus and the cytoplasm. The clear cell type showed no nuclear staining but demonstrated an unusual deeply stained speckled pattern in the cytoplasm (Table 1). Representative photomicrographs were taken and are shown in Figure 4.

**Table 1:** Pattern of hRad17 staining by ovarian cancer subtype

Ovarian cancer subtype	Number of cases	hRad17 staining	Percent over-expressing hRad17
Benign	9	Nuclear	13%
Serous	72	Nuclear and cytoplasmic	42%
Endometrioid	19	Cytoplasmic	47%
Clear cell	10	Cytoplasmic	80%
Mucinous	6	Nuclear, cytoplasmic, and stromal	100%

### Patient Survival Analysis

**Figure 5:**
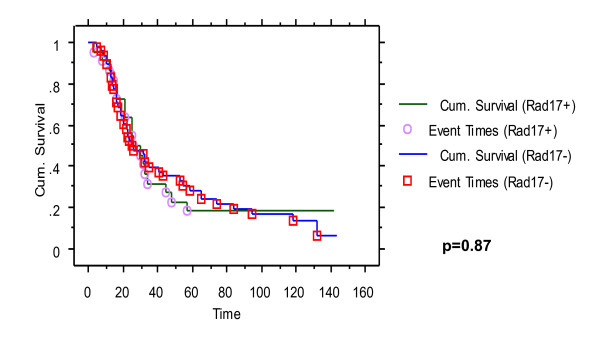
**Kaplan-Meier Curve demonstrating no difference in survival of patients with Stage III C ovarian cancer comparing hRad17 positive tumors with hRad17 negative tumors**.

Patient survival data was collected on the 72 Grade 3, Stage IIIC serous cancers that were stained for hRad17. Kaplain-Meier curves were generated to compare hRad17 over-expression to patient survival (Figure 5). There was no significant difference of hRad17 expression when comparing short-term survivors to long-term survivors with Stage IIIC, Grade 3/3 serous ovarian cancer. When patients were further divided into optimally and suboptimally debulked (as defined by <2 cm residual disease at the completion of surgery) there was still no correlation found between hRad17 over-expression and patient survival (data not shown).

## Discussion

Bao et al. initially cloned hRad17 after discovering a unique protein that was differentially expressed in colon cancer over normal colonic tissue[7]. hRad17 is over-expressed in 54.7% of all breast cancers and in 68% of those breast cancers with lymph node metastases[24]. Similarly, comparison of non-small cell lung cancer to normal tissue has shown overexpression of hRad17[14] and demonstrated a significant correlation of hRad17 expression with the presence of lymph node metastases[27]. In contrast, hRad17 is down-regulated in head and neck cancers compared to normal oral mucosa likely secondary to gene deletion, possibly contributing to increased rates of DNA mutations in head and neck tumors[28].

Our study found that hRad17 is over-expressed in ovarian cancer, as seen in breast, colon, and lung cancer. Further, the pattern of expression differs among subtypes of ovarian epithelial cancers. Unlike studies in breast and lung cancer that found over-expression correlated with metastases, we did not find that hRad17 over-expression correlated with survival in a subgroup of patients with Stage IIIC serous ovarian cancer. Given this protein's role as in the cell cycle checkpoint, upregulation of hRad17 may increase a tumor's resistance to platinum agents which rely on DNA damage for cell death. This hypothesis would need to be tested in a larger group of patients with mucinous and clear cell tumors. Alternatively, over-expression may represent accumulation of a nonfunctional protein. Future studies including staining the tissue sections with Ki67 may help to elucidate this unusual staining pattern. We would hypothesize that Ki67 overexpression would be more likely in tissues showing predominately nuclear staining.

Other studies have suggested that abnormal expression or distribution of hRad17 may lead to a loss of function as a DNA damage repair protein. hRad17 has been characterized as a nucleolar protein when functional in DNA repair. We found differential expression of this protein in both the cytoplasm and nucleus depending on the histologic type. Dispersion of hRad17 may correlate with a loss of function as a DNA repair protein. Functional studies are needed to characterize the role of hRad17 in these tumors, both in the nucleus and the cytoplasm.

This study consisted of a small sample set for comparison of survival data and a larger number of cases may show a significant difference in expression between short-term and long-term survivors. Further, the use of immunohistochemistry to determine expression for survival comparison may be insufficiently quantitative to see a significant difference. Limitations in the amount of tissue did not allow for direct RNA or protein measurement in each tumor. Lastly, these data do not give any information about the role of hRad17 in ovarian cancer.

## Conclusions

hRad17 is over-expressed in a majority of epithelial ovarian cancers. Furthermore, this over-expression varies by subtype suggesting a role in the pathogenesis and behavior of these types. Functional studies are needed to determine the potential role of this protein in the development of ovarian cancer.

## List of Abbreviations used

cDNA: complimentary DNA; HOSE: human ovarian surface epithelium cell line; RT-PCR: real time polymerase chain reaction; GAPDH: Glyceraldehyde 3-phosphate dehydrogenase; SDS-PAGE: sodium dodecyl sulfate polyacrylamide gel electrophoresis; PVDF: polyvinylidene fluoride; TBST: Tris-buffered saline Tween-20; TBS: Tris-buffered saline; BSA: Bovine serum albumin

## Competing interests

The authors declare that they have no competing interests.

## Authors' contributions

JY performed the immunohistochemistry, western blot analysis, data analysis, and drafted the manuscript. CK participated in the acquisition of samples, clinical data analysis, western blot analysis, and RT-PCR. JK participated in the acquisition of samples, western blot analysis, and RT-PCR. WW reviewed all of the slides for pathologic confirmation of cell type and provided all of the samples. MM participated in study design, acquisition of samples, and acquisition of clinical data. RB participated in study design, acquisition of samples, and acquisition of clinical data. SM conceived of the study design, participated in immunohistochemistry, western blot, data analysis, and manuscript writing. All authors critically reviewed drafts of the manuscript and have approved the final version for publication.
